# Acupuncture in the real world: evaluating a 15-year NADA auricular acupuncture service for breast cancer survivors experiencing hot flushes and night sweats as a consequence of adjuvant hormonal therapies

**DOI:** 10.1007/s00520-022-06898-7

**Published:** 2022-02-26

**Authors:** Beverley de Valois, Teresa Young, Pam Thorpe, Tarsem Degun, Karen Corbishley

**Affiliations:** 1 Supportive Oncology Research Team, East and North Hertfordshire NHS Trust Incorporating Mount Vernon Cancer Centre, Rickmansworth Road, Northwood, HA6 2RN Middlesex UK; 2 Centre for Academic Primary Care, Population Health Sciences, Bristol Medical School, University of Bristol, 39 Whatley Road, Bristol, BS8 2PS UK; 3 Lynda Jackson Macmillan Centre, Mount Vernon Cancer Centre, Rickmansworth Road, Northwood, HA6 2RN Middlesex UK

**Keywords:** Acupuncture (MeSH), Auricular acupuncture (MeSH), Breast cancer (MeSH), Hot flushes (MeSH), Cancer Survivors (MeSH), Menopause (MeSH)

## Abstract

**Purpose:**

While clinical trials provide valuable data about efficacy of interventions, findings often do not translate into clinical settings. We report real world clinical outcomes of a 15-year service offering breast cancer survivors auricular acupuncture to manage hot flushes and night sweats (HFNS) associated with adjuvant hormonal treatments. This service evaluation aims to (1) assess whether usual practice alleviates symptoms in a clinically meaningful way and (2) compare these results with scientific evidence.

**Methods:**

Data were analysed from 415 referrals to a service offering women eight standardised treatments using the National Acupuncture Detoxification Association (NADA) protocol. Outcome measures administered at baseline, end of treatment (EOT), and 4 and 18 weeks after EOT included hot flush diaries, hot flush rating scale (HFRS) and women’s health questionnaire (WHQ).

**Results:**

Over 2285 treatments were given to 300 women; 275 (92.3%) completed all eight treatments. Median daily frequency of HFNS reduced from 9.6 (IQR 7.3) to 5.7 (IQR 5.8) at EOT and 6.3 (IQR 6.5) 18 weeks after EOT. HFRS problem rating showed a clinically meaningful reduction of ≥ 2 points at all measurement points. WHQ showed improvements in several symptoms associated with the menopause. Two adverse events were reported, neither were serious. Results are comparable to published research.

**Conclusion:**

This first analysis of a long-term auricular acupuncture service compares favourably with outcomes of other studies for reducing HFNS frequency and associated menopausal symptoms. In day-to-day clinical practice, NADA appears to be a safe effective intervention for breast cancer survivors.

## Background

Hot flushes and night sweats (HFNS) are a consequence of adjuvant hormonal treatments (tamoxifen and aromatase inhibitors) for breast cancer [1], of which there were 55,000 new cases annually in the UK from 2015–2017 [2]. They have been reported by up to 84% of women taking tamoxifen, with 60% reporting symptoms as severe [3]. Previous opinion held that these symptoms alleviate with time [4]; however, recent research reports that prevalence may remain static for the duration of adjuvant hormonal treatment and continue for 5 years after treatment ends [3, 5].

A common symptom of natural menopause, hot flushes are described as sudden sensations of heat, often accompanied by sweating, reddening of the skin, and sometimes by palpitations [6]. They are associated with impaired quality of life, poor sleep, and negative mood [7], with night sweats associated with a greater risk of minor depression, fatigue and mood changes [8].

Cancer treatment induced HFNS are reported as more severe, frequent, and longer-lasting than those of natural menopause [1, 9]. HFNS have a wider implication for cancer survival, as treatment side effects are cited as a key reason for low adherence to endocrine therapy, with consequent impact on survival [10].

The main treatment for menopausal symptoms, hormone replacement therapy (HRT), is contraindicated for use with women with breast cancer, as are progestogens [1, 8]. Non-hormonal pharmacological treatments most likely to be offered for HFNS management include selective serotonin (and norepinephrine) reuptake inhibitors (SSRIs and SNRIs), especially venlafaxine, as well as gabapentin and clonidine. Due to concerns about the inhibitory effects of SSRIs and SRNIs on tamoxifen, paroxetine and fluoxetine are not recommended for women taking tamoxifen [1]. However, some women who are prescribed these medications do not find them helpful, whilst others find the mild to moderate beneficial effects are outweighed by their adverse effects [1, 5]. Furthermore, many women do not want to take medications in addition to their adjuvant hormonal therapy and seek non-pharmacological options [9, 11].

Acupuncture is a non-pharmacological intervention considered by healthcare professionals and breast cancer survivors alike. A survey of UK healthcare professionals reported 50% suggested it to breast cancer survivors experiencing HFNS, with over 50% of those women who used it reporting it as being helpful [5]. As an intervention for managing HFNS, it has been widely researched [12]. In RCTs comparing acupuncture with pharmacological interventions, acupuncture was found to be equally effective as gabapentin [13] and venlafaxine [14], with less rebound effect after the end of active treatment and fewer adverse effects [15]. In addition, acupuncture appears to have beneficial effects on the wider symptoms associated with menopause [16], including sleep problems, anxiety, low mood, poor concentration, as well as improved energy and health-related quality of life. In examining longer term benefits, systematic reviews report that the maintenance effect on HFNS and associated menopausal symptoms is at least 3 months, suggesting that acupuncture’s effects are not short term or placebo [17, 18]. Furthermore, the reported side effects are minor and are typically bruising or transient pain at the needling site [14, 19–24].

While systematic reviews of acupuncture generally conclude that methodological issues compromise the findings of RCTs, it is also known that the findings of RCTs often do not translate to clinical settings [25]. We evaluated real-world clinical outcomes for breast cancer survivors receiving standardised auricular acupuncture, the NADA (National Acupuncture Detoxification Association) protocol [26, 27], for HFNS by evaluating data collected from a 15-year service offered in a hospital outpatient setting. The aims were to:

- Provide an insight into whether usual clinical practice can alleviate symptoms to a clinically meaningful extent.
- Assess whether real-world acupuncture data aligns with scientific evidence.

In reporting this evaluation, this paper adheres to the Template for Intervention Description and Replication (TIDierR) Checklist [28].

## Materials and methods

### Approval and setting

The NADA acupuncture service was offered from 2005 to 2020 by the Complementary Therapy Service (CTS) at the Lynda Jackson Macmillan Centre (LJMC), a cancer information and drop-in centre associated with Mount Vernon Cancer Centre in Northwood, Middlesex UK. Data collection to evaluate the service was approved by the LJMC Medical Director in 2005; in line with the Declaration of Helsinki guidelines for unproven interventions in clinical practice, all service users gave written consent to their participation in the evaluation of the service [29]. The CTS was supported by the Centre’s research team, who administered and analysed the service data.

UK non-acupuncturists with a healthcare qualification (including integrative practitioners) can become NADA specialists on receiving initial training and annual updates. This service saw four practitioners train as NADA specialists, one of whom was a specialist breast cancer nurse, and the others were integrative therapists practising a range of therapies. Co-authors PT and TD have further developed the service and administered treatments for the last 13 and 8 years respectively.

### Service description

This service was built on our previous research using the NADA protocol, which we have reported elsewhere [22, 30]. Briefly, the research and service comprise an intake interview at least 2 weeks prior to the first NADA treatment, then a course of eight treatments delivered weekly in a group setting to women meeting these criteria: taking adjuvant hormonal treatments for early breast cancer (tamoxifen or aromatase inhibitors) for ≥ 6 months, active cancer treatment (surgery, chemotherapy and/or radiotherapy) completed ≥ 6 months previously, self-reporting an average of ≥ 4 HFNS in a 24-h period for ≥ 3 months, and not receiving concurrent complementary therapies. Women were referred by their oncology healthcare professionals or could self-refer.

Once established, the service evolved to fulfil the Centre’s ethos of care, adapting in response to the needs of women who were service users rather than research participants. Changes include the following:

• A sample treatment (needles in one ear for 5 min) was given at the intake interview after the woman gave written consent for treatment and completed baseline measures.
• Increased focus on developing the therapeutic relationship, which had been minimised in the research.
  • Therapists offered support when administering the baseline outcome measures, as some women found completing these challenging.
• Groups were limited to four women, rather than five, in response to feedback about the comfort of the small clinical spaces available in the centre.
• A brief group relaxation exercise was introduced prior to needling.
• Needling took place in the group, rather than individually in a separate room, as was done in the research.

Other changes related to data collection. Mid-treatment outcomes were not monitored, reducing the number of measurement points to four, as described below. The NADA specialists administered and collected the outcome measures at baseline. At the eighth and final treatment, they gave the women the end of treatment outcome measures and a stamped addressed envelope, with instructions to complete them that day. These were to be posted with the completed hot flush diary 2 weeks later to the research team’s data administrator, who also administered the outcome measures for the two follow-up points by post.

Safety and adverse events were formally recorded on incident forms and in patient notes following procedures applied to all complementary therapy services delivered in the LJMC.

### Outcome measures

To monitor the service, we used the same validated outcome measures as in our previous research. These were administered at baseline, end of treatment (EOT), and at four (post-tx 4) and 18 (post-tx 18) weeks after EOT. Briefly outlined below, we have described these elsewhere [21, 22]:

• Hot flush diaries – to measure HFNS frequency and severity these in-house designed booklets enable women to record frequency and severity of incidents at 2-hourly intervals daily, ideally as they occur, for up to 14 days.
• Hot flush rating scale (HFRS) – to measure the extent to which HFNS are a problem, this validated self-report measure has three questions using 10-point Likert scales to rate the extent to which HFNS are problematic, distressing, and the cause of interference in daily life [31, 32]. Problem rating is calculated as the mean of the three questions, with higher scores indicating more problematic HFNS. A change of 2 points on this scale is considered clinically relevant [33].
• Women’s health questionnaire (WHQ) – to evaluate quality of life, this validated 36-item questionnaire measures changes in nine domains of physical and emotional health associated with the menopause transition. Analysis according to the *WHQ Women’s Health Questionnaire User Guide* [34] results in a score between 0.00 and 1.00, where higher scores are associated with worse symptomology and quality of life. A change of 0.10–0.20 is clinically meaningful.

### Statistics

Data were analysed using SPSS V22.0. Diaries were designed for recording frequency per day, with each day divided into 12 2-hourly intervals. Women could input “0” for any 2 h in which they experienced no HFNS. Any empty cells were considered “missing data”. Diaries were visually inspected to check that any missing data were random (for example, consistently not reporting at the weekend, or during time periods such as the afternoon). Provided at least six of the 12 2-hourly intervals were completed on any 1 day (including “0” for no HFNS) and any missing data appeared to be random, the daily means and medians were calculated from the available data (effectively imputing missing data points with the daily means or medians calculated from the reported data points).

As in our previous HFNS studies, log transformation of these calculated daily means produced a normal distribution, allowing parametric *t* tests to be used for comparison with baseline scores [21, 22].

Data from the HFRS and WHQ were assessed for normality. On the WHQ domains Depressed Mood and Memory/Concentration were very skewed, whilst other scales were less skewed but exhibited kurtosis. Only Somatic Symptoms was normally distributed. Data were therefore analysed using both Student’s *t* tests and their non-parametric equivalent. Similar significance levels were achieved with both analyses, so only *t* tests are reported here.

## Results

We are interested in both short- and long-term results, and thus we report data from all measurement points.

### Enrolment, attendance, and questionnaire return

**Fig. 1:**
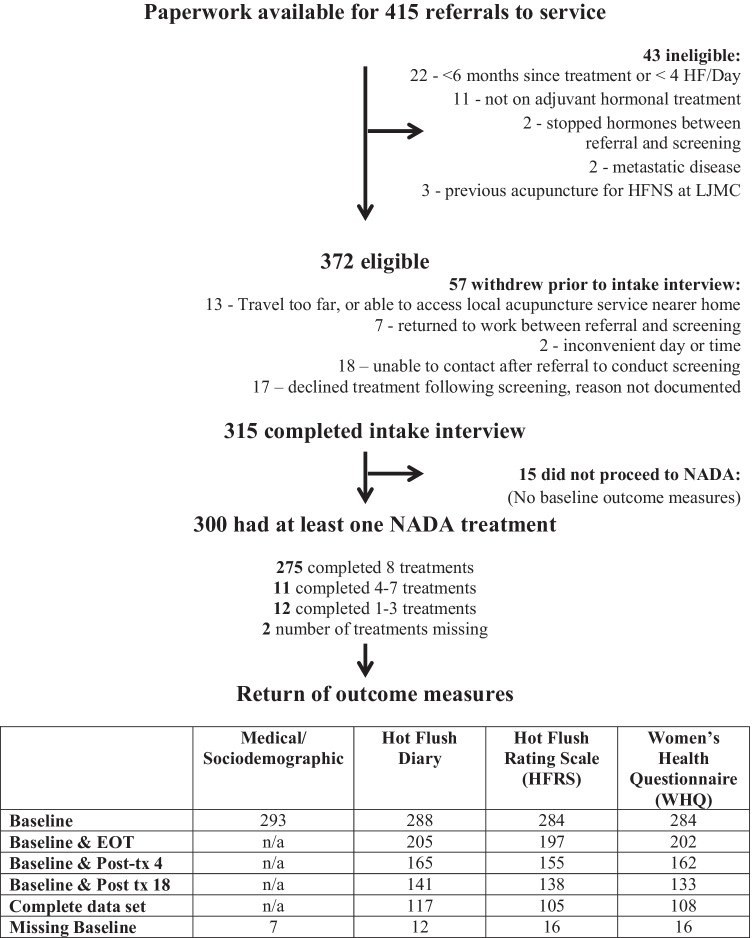
Flow diagram showing progress of 415 referrals including eligibility, treatment, and return of outcome measures

Figure 1 displays the course of 415 referrals to the service and the resulting data sets used in this evaluation, including reasons for ineligibility, withdrawal prior to treatment, treatments received, and data return.

At least 2285 NADA treatments were given to 300 women, of whom 275 (92.3%) completed the full course of eight treatments, with a mean of 7.6 and a median of 8 treatments per individual. Questionnaire return at EOT was 71% and 49% at 18 weeks post-treatment. Forty percent of the women returned all questionnaires at all time points. There was no significant difference in baseline scores on any of the outcome measures between participants completing only baseline measures and those completing baseline and at least one set of follow-up measures.

### Participant demographics and clinical features

Key features of the baseline characteristics (Table 1) are a predominately white British ethnicity (74%), the majority of whom were currently taking tamoxifen (63.7%) followed by anastrozole (15.7%). Nearly 70% of the women had been receiving adjuvant hormone therapy for 6 to 24 months.

**Table 1:** Demographic and clinical details at baseline (*n* = 300)

Age	
Mean (median) Range Missing	53 (52) 33—78 6
**Demographic characteristics**	**n (%)**
**Marital status**	
Single Married Re-married Living with partner Separated Divorced Widowed Missing	25 (8.3) 165 (55) 34 (11.3) 24 (8) 9 (3) 21 (7) 15 (5) 7 (2.3)
**Ethnic background**	
Asian—other Black (African, British, Caribbean) Indian Pakistani White British White other Other Missing	5 (1.6) 10 (3.3) 17 (5.7) 3 (1) 222 (74) 20 (6.7) 6 (2) 17 (5.7)
**Education**	
Less than compulsory Compulsory Post compulsory University Post-graduate Missing	22 (7.3) 113 (37.7) 68 (22.7) 53 (17.7) 28 (9.3) 16 (5.3)
**Current employment status**	
Retired Not working Working part-time Working full-time Missing	64 (21.) 51 (17) 100 (33.3) 76 (25.3) 9 (3)
**Clinical characteristics**	
**Current adjuvant hormonal therapy (AHT)**	
Anastrozole Exemestane Letrozole Tamoxifen AHT plus Zoladex Other Missing	47 (15.7) 13 (4.3) 25 (8.3) 191 (63.7) 5 (1.7) 1 (0.3) 18 (6)
**Time receiving adjuvant hormonal therapy (AHT)**	
< 6 months 6–12 months 1–2 years 2–3 years ≥ 3 years Missing	30 (10) 130 (43.3) 78 (26) 19 (6.3) 9 (3) 34 (11.3)
**Cancer treatment history**	
**Surgery**	
Mastectomy Wide local excision Other No surgery Missing	106 (35.5) 171 (57) 2 (0.6) 2 (0.6) 19 (6.3)
**Radiotherapy**	
Radiotherapy—yes Radiotherapy—no Missing	164 (54.7) 36 (12) 100 (33.3)
**Chemotherapy**	
Neo-adjuvant—yes Neo-adjuvant—no Neo-adjuvant—missing Chemotherapy adjuvant—yes Chemotherapy adjuvant—no Chemotherapy adjuvant —missing	34 (11.3) 238 (79.3) 28 (9.3) 122 (40.7) 142 (47.3) 36 (12)
**Menopausal status**	
Perimenopause (last period within previous year) Menopause (no period within previous 1–5 years) Postmenopausal (no period in > 5 years) Missing	36 (12) 86 (28.7) 48 (16) 130 (43.3)
**History of HRT**	
Have taken HRT Have not taken HRT Missing	91(30.3) 187 (62.3) 22 (7.3)
**Co-morbidities self-reported by patients**	
Number of co-morbidities	
0 1 2 3 4 5 6–11	78 (26) 81 (27) 68 (22.7) 31 (10.3) 20 (6.7) 11 (3.7) 11 (3.7)
Co-morbidities*	
Allergy Anaemia Asthma Cardiovascular other than hypertension Depression Diabetes Digestive diseases Genitourinary disorders Musculo-skeletal Respiratory	42 (14) 8 (2.7) 32 (10.7) 7 (2.3) 54 (18) 16 (5.3) 25 (8.3) 11 (3.7) 80 (26.7) 5 (1.7)

^*^Other co-morbidities reported include fatigue, anxiety, Crohn’s disease, hypothyroidism

### HFNS frequency

At baseline, women were experiencing a median of 9.6 (interquartile range [IQR] 7.3) HFNS per day, reducing to 5.7 (IQR 5.8) at EOT, and 6.3 (IQR 6.5) at 18 weeks after EOT (Table 2).

**Table 2:** NADA HFNS frequency (per day)

	Baseline *n* = 205/300	EOT *n* = 205/300	Post-tx 4 *n* = 166/300	Post-tx 18 *n* = 140/300
Mean (standard deviation)	10.9 (8.1)	6.9 (5.0)	6.8 (4.9)	7.2 (5.0)
95% confidence interval	9.8–12.0	6.2–7.6	6.0–7.5	6.4–8.1
Median (inter quartile range)	9.6 (7.3)	5.7 (5.8)	5.6 (6.1)	6.3 (6.5)
Minimum mean score	1.57	0	0	0
Maximum mean score	94.3	29	24.4	22.7
Range	92.73	29	24.4	22.7

Analysis revealed that 7% of women had fewer HFNS than the ≥ 4 per 24-h period stated in the eligibility criteria. HFNS counts were self-reported at the intake interview and could not be verified until the baseline hot flush diary was returned on the day of the first NADA treatment and subsequently analysed. This suggests that women found even low numbers of HFNS problematic enough to warrant having treatment.

At the other end of the spectrum, 10% recorded 24–36 HFNS per day and one woman reported as many as 94 HFNS in a day. Although this could be considered an outlier, we have included it in the analysis as an example of how extreme HFNS can be.

### Hot flush rating scale

At baseline, women were experiencing a mean problem rating score of 7.56 (SD 1.7) out of 10, reducing to 5.42 (2.55), 4.90 (2.53), and 5.18 (2.56) at EOT, 4 weeks post-EOT and final follow-up at 18 weeks. (Table 3).

**Table 3:** Changes in HFRS and WHQ at four time points

	Baseline (*n* = 202)	EOT (*n* = 202)		Baseline (*n* = 162)	Post-tx 4 (*n* = 162)		Baseline (*n* = 133)	Post-tx 18 (*n* = 133)	
	**Mean (SD)**	**Mean (SD)**	**∆Baseline–EOT**	**Mean (SD)**	**Mean** **(SD)**	**∆Baseline–post-Tx 4**	**Mean** **(SD)**	**Mean** **(SD)**	**∆Baseline–post-Tx 18**
**HFRS**	7.56 (1.70)	5.42 (2.55)	**2.14**	7.37 (1.72)	4.90 (2.53)	**2.47**	7.37 (1.71)	5.18 (2.56)	**2.19**
**WHQ domain**									
Anxiety/fears (ANX)	0.39 (0.33)	0.27 (0.27)	**0.12**	0.38 (0.32)	0.23 (0.28)	**0.15**	0.38 (0.33)	0.28 (0.30)	**0.10**
Attractiveness (ATT)	0.61 (0.29)	0.65 (0.31)	-0.04	0.63 (0.29)	0.66 (0.31)	− 0.03	0.63 (0.29)	0.66 (0.32)	-0.03
Depressed mood (DEP)	0.31(0.17)	0.25 (0.14)	0.06	0.30 (0.18)	0.27 (0.16)	0.03	0.29 (0.17)	0.27 (0.16)	0.02
Memory/concentration (MEM)	0.64(0.36)	0.57 (0.36)	0.07	0.63 (0.36)	0.60 (0.38)	0.03	0.65 (0.36)	0.61 (0.36)	0.04
Menstrual symptoms (MEN)	0.34 (0.24)	0.30 (0.24)	0.04	0.34 (0.24)	0.29 (0.24)	0.05	0.33 (0.25)	0.31 (0.25)	0.02
Sexual behaviour(SEX) (*n* = 123 baseline pairs)*	0.49 (0.32)	0.44 (0.37)	0.05	0.48 (0.32)	0.43 (0.33)	0.05	0.48 (0.31)	0.44 (0.32)	0.04
Sleep problems (SLE)	0.64 (0.29)	0.48 (0.33)	**0.16**	0.62 (0.29)	0.51 (0.32)	**0.11**	0.64 (0.30)	0.58 (0.34)	0.06
Somatic symptoms(SOM)	0.52 (0.23)	0.43 (0.23)	0.09	0.51 (0.23)	0.47 (0.25)	0.04	0.50 (0.23)	0.47 (0.25)	0.03
Vasomotor symptoms (VAS)	0.97 (0.14)	0.79 (0.33)	**0.18**	0.97 (0.14)	0.79 (0.33)	**0.18**	0.96 (0.15)	0.86 (0.30)	**0.10**

Higher scores are associated with worse symptomology and quality of life. Clinically significant changes are highlighted in **bold. HFRS:** a change of 2 points is considered clinically meaningful. **WHQ:** a change of 0.10–0.20 is clinically meaningful. **Sexual behaviour (SEX):** only 123 patients completed baseline and EOT (not 202 as for other scales), falling to 100 and 82 at 4 and 18 weeks respectively

### Women’s health questionnaire

Improvements were seen in all but one domain at all time points (Table 3), compared to baseline. These were clinically significant (a change of ≥ 0.1) for anxiety/fears, sleep problems, and vasomotor symptoms.

### Comparing NADA service outcomes with our previous NADA research

Comparisons of the outcomes of the service with our previous NADA research are shown in Table 4. Paired ‘*t*’ tests show significant improvements for both service users (SU) and research participants (RP) for HFNS frequency, HFRS, and WHQ domains.

**Table 4:** Comparison of NADA research participants (RP) with NADA service users (SU): mean changes in outcome measures

	Time period baseline to:	Group	*n* =	Change	Lower (5%)	Upper (95%)	‘*t*’ (paired)	*p* <	‘*t*’ (unpaired)	*p*
**HFNS frequency**										
Mean % reduction	EOT	RP SU	47 205	35.9% 44.4%	25.4% 38.7%	45.4% 49.5%	5.8 12.0	0.0001 0.0001	1.5	NS
	Post-tx 4	RP SU	45 166	37.5% 48.2%	25.4% 41.7%	47.6% 54.0%	5.3 11.1	0.0001 0.0001	1.8	NS
	Post-tx 18	RP SU	38 141	37.1% 46.7%	24.8% 37.4%	47.4% 54.6%	5.3 7.8	0.0001 0.0001	1.4	NS
**Hot flush rating scale (HFRS)**										
	EOT	RP SU	48 197	2.15 2.14	1.55 1.80	2.74 2.48	7.2 12.3	0.0001 0.0001	0.9	NS
	Post-tx 4	RP SU	46 155	2.17 2.47	1.59 2.07	2.75 2.87	7.5 12.26	0.0001 0.0001	2.1	**0.04**
	Post-tx 18	RP SU	31 138	1.81 2.19	1.17 1.76	2.44 2.63	5.8 11.4	0.0001 0.0001	2.2	**0.03**
**Women’s health questionnaire (WHQ)**										
**ANX**	EOT	RP SU	47 202	.10 .12	.03 .07	.17 .16	2.9 5.4	0.006 0.0001	0.4	NS
	Post-tx 4	RP SU	45 162	.09 .15	.01 .11	.17 .19	2.3 7.0	0.03 0.0001	1.4	NS
	Post-tx 18	RP SU	39 133	.06 .10	− .02 .05	.15 .14	1.5 4.1	0.2 0.0001	0.8	NS
**ATT**	EOT	RP SU	44 193	.06 − .04	− .04 − .08	.15 .01	1.2 − 1.6	0.3 0.2	− 1.9	NS
	Post-tx 4	RP SU	44 154	.07 − .03	− .04 − .09	.18 .02	1.2 − 1.2	0.3 0.3	− 1.7	NS
	Post-tx 18	RP SU	37 124	.01 − .03	− .09 .09	.12 .03	.3 − .9	0.8 0.4	− 0.7	NS
**DEP**	EOT	RP SU	47 202	.15 .06	.08 .03	.22 .08	4.1 4.3	0.0001 0.0001	** − 2.5**	**0.03**
	Post-tx 4	RP SU	44 162	.09 .03	.03 .00	.15 .06	2.9 2.0	0.006 0.05	− 1.7	NS
	Post-tx 18	RP SU	39 133	.02 .02	− .06 − .01	.09 .05	.4 1.4	0.7 0.2	0.06	NS
**MEM**	EOT	RP SU	47 202	.12 .07	.02 .03	.22 .12	2.4 3.2	0.03 0.001	− 0.8	NS
	Post-tx 4	RP SU	46 162	.08 .03	− .02 − .02	.18 .09	1.7 1.3	0.1 0.2	− 0.8	NS
	Post-tx 18	RP SU	39 131	.13 .04	.01 − .01	.24 .10	2.2 1.6	0.04 0.2	− 1.4	NS
**MEN**	EOT	RP SU	48 198	.06 .04	.00 .01	.12 .07	2.0 2.3	0.06 0.02	− 0.6	NS
	Post-tx 4	RP SU	46 158	.05 .05	− .01 .01	.11 .09	1.9 2.6	0.07 0.01	− 0.03	NS
	Post-tx 18	RP SU	40 133	.04 .02	− .03 − .02	.12 .06	1.2 .8	0.30 0.5	− 0.06	NS
**SEX**	EOT	RP SU	34 123	.08 .05	− .01 − .01	.17 .10	1.8 1.8	0.09 0.08	− 0.06	NS
	Post-tx 4	RP SU	32 100	.03 .05	− .05 .00	.11 .09	0.8 1.9	0.5 0.06	− 0.4	NS
	Post-tx 18	RP SU	26 82	.06 .04	− .01 − .02	.14 .10	1.7 1.3	0.1 0.3	− 0.4	NS
**SLE**	EOT	RP SU	48 202	.22 .16	.10 .11	.31 .21	4.9 6.5	0.0001 .0001	− 1.1	NS
	Post-tx 4	RP SU	46 162	.13 .11	.05 .06	.21 .17	3.2 4.0	0.003 0.0001	− 0.4	NS
	Post-tx 18	RP SU	40 133	.11 .06	.01 − .01	.21 .12	2.1 1.8	0.05 0.08	− 0.9	NS
**SOM**	EOT	RP SU	47 200	.12 .09	.06 .06	.19 .12	3.7 5.8	0.001 0.0001	− 0.7	NS
	Post-tx 4	RP SU	45 161	.07 .04	.00 .01	.13 .08	2.1 2.3	0.04 0.02	− 0.8	NS
	Post-tx 18	RP SU	39 136	.04 .03	− .04 − .01	.12 .06	1.1 1.6	0.3 0.2	− 0.3	NS
**VAS**	EOT	RP SU	46 199	.23 .18	.12 .13	.34 .11	4.3 7.5	0.0001 0.0001	− 0.9	NS
	Post-tx 4	RP SU	44 160	.14 .18	.05 .12	.23 .23	3.0 6.7	0.003 0.0001	0.7	NS
	Post-tx 18	RP SU	39 136	.14 .10	.04 .04	.24 .15	2.9 3.6	0.006 0.0001	− 0.7	NS

For the WHQ and HFRS, negative changes denote deterioration in symptomology and quality of life

*RP*, **research participants;** *SU*, **service users;** *EOT*, **end of treatment;** *Post-tx 4*, **4 weeks after EOT; *Post-tx 18*, 18 weeks after EOT;** *NS*, **not significant**; *ANX*, **anxiety/fears;** *ATT*, **attractiveness;** *DEP*, **depressed mood;** *MEM*, **memory/concentration;** *MEN*, **menstrual symptoms;** *SEX*, **sexual behaviour;** *SLE*, **sleep problems;** *SOM*, **somatic symptoms;** *VAS*, **vasomotor symptoms**

Unpaired ‘*t*’ tests show no significant differences between SU and RP with the exception of HFRS, where SU had greater improvements than RP at both follow-up points. Additionally, RPs had greater improvements on the WHQ depressed mood domain.

### Safety and adverse events

There were two recorded incidents of adverse reactions. Dizziness experienced at the first treatment caused one woman to immediately discontinue NADA treatment, while another woman attributed dizziness and nausea on initial needling to an existing migraine and continued all further treatments.

Pain at the needle site and bleeding on removal of needles were experienced by some women; these were managed according to NADA protocol, recorded in the patient notes, and are not analysed here. These are all commonly reported as minor adverse events of acupuncture [35].

One incident form recorded a lost needle. The potential for losing needles was diminished when the design of needle handles changed from stainless steel to fluorescent orange plastic, making them easy to locate if they fell out of the ear during treatment.

## Discussion

The aims of this evaluation were to gain insight into whether usual clinical practice of acupuncture can alleviate the symptoms associated with adjuvant hormonal treatments for early breast cancer in a clinically meaningful way. This is, to our knowledge, the first evaluation of a real world long running service and provides an opportunity to assess whether results align with scientific evidence.

We acknowledge many limitations to this evaluation. Despite its longevity, the numbers of women treated are small. While NADA is often delivered in groups of up to 20 or more (Rachel Peckham, ‘The role and the impact of the NADA protocol’, unpublished MSc thesis, University of Westminster, 2005) the small size of the available treatment rooms limited group size. Additionally, fully supported resources, such as dedicated funding, were not in place in the early years of the service. Full management support was eventually garnered as the service established a reputation and consistently received referrals from oncologists and self-referral.

Incompleteness of the data is another limitation. A service is not a research study and service users’ expectations of using a service differ from those of participants in a research study. Although therapists stressed the importance of returning questionnaires for monitoring the service, it was not necessarily a priority for service users to do this, as illustrated by the returns at EOT, 4 and 18 weeks (71%, 57%, and 49%). In the research study, 48/51 (94%) of women returned both baseline and EOT questionnaires, falling to 90% and 78% at 4 and 18 weeks follow-up. Longevity of the service is another factor, as some data (mostly documents recording referrals for women who then did not commence treatment) were lost in office refurbishments.

Nevertheless, the data offer many interesting insights. Women adhered to treatment, with 92% completing a full course of eight treatments. This suggests that the intervention was acceptable and that women experienced sufficient benefit to invest time in travelling to the hospital and having treatment. NADA acupuncture was also safe with only two formally recorded adverse events in over 2285 treatments.

### HFNS frequency

HFNS frequency analysis shows a significant reduction at EOT, and this 44% (*p* < 0.0001) reduction compares favourably with EOT results of other research studies. Walker et al. reported a 50% (*p* < 0.001) reduction after 16 body acupuncture treatments over 12 weeks, a reduction reported as equivalent to venlafaxine [14]. A systematic review of six randomised controlled trials (RCTs) by Frisk et al. reported a mean reduction of 43.2% from baseline to end of acupuncture (range 5–12 weeks of treatment, *n* = 172) [18]. These comparisons raise the question of dose. Our dosage of eight treatments over 8 weeks lies at the lower end of these scales; it would be interesting to compare the results of NADA following an extended treatment regimen.

We note that our inclusion criterion of ≥ 4 HFNS per 24-h period for ≥ 3 months is higher than that used for most studies of HFNS. (We also note that despite 7% of women reporting fewer than this, the median number of HFNS at baseline was 9.6 (IQR 7.3) per day). A systematic review of RCTs of active interventions for HFNS reports entry criteria from as few as four HFNS per week for 1 month, with nine of the 13 studies reported set at ≥ 2 per day [36]. Our criteria may be classified as “high frequency”, defined as ≥ 12 hot flushes and ≥ 3 night sweats a week [7].

Our follow-up data suggest that improvement was maintained from EOT to 18 weeks. This 46.7% (*p* < 0.0001) reduction again compares favourably with Frisk’s reported 45.6% at final follow-up (mean 6 months, range 3–9, *n* = 153) [18]. However, our result must be interpreted with caution due to missing data. It is unclear whether women were not motivated to return hot flush diaries because they did not experience benefit, or whether HFNS were no longer troublesome enough to warrant completing the paperwork.

### HFNS as a problem

There is debate in the literature as to whether frequency counts are the most appropriate measure of HFNS, as frequency alone may not accurately assess the impact of HFNS on a woman’s life [6, 7]. While frequency is often the main outcome in assessments of treatment, reducing the interference of HFNS and improving quality of life may be more meaningful measures of a successful intervention [7].

The HFRS captures the impact of HFNS on women’s lives and on their quality of life [32]. NADA service HFRS data show clinically and statistically significant improvement in HFNS as a problem in both the short and long term. We compared service results with HFRS results from an RCT comparing nurse-led cognitive behavioural therapy (CBT) with usual care for breast cancer–related HFNS (Table 5) [37]. NADA maintained a clinically meaningful reduction of ≥ 2 points at all measurement points. While it appears to have performed less well than CBT (− 2.4 points compared to − 3.2 points at final follow-up), it appears to be superior to usual care (− 1 point at final follow-up).

**Table 5:** Comparison of NADA service HFNS frequency and HFRS problem rating scores with Fenlon et al. CBT study [1]

HFNS frequency (per week)			
	NADA (median IQR, *n*)	CBT (median IQR) *n* = 63	Usual care (median IQR) *n* = 67
Baseline	67.0 (37.9–96.1, 205)	58.0 (35.0–84.0)	63.0 (28.0–91.0)
EOT^1^	40.0 (0–80.4, 205)	38.5 (16.0–73.0)	49.0 (22.0–80.5)
Post-tx 4	38.0 (0–82.9, 155)	n/a	n/a
Final follow-up^2^	44.0 (0–88.1, 133)	42.0 (17.0–63.0)	56.0 (28.0–77.0)
Change: final follow-up minus baseline	− 23.0	− 16.0	− 7.0
**Hot flush rating scale (HFRS)**			
**HFRS scores**	NADA (mean SD, *n*)	CBT (mean SD) *n* = 63	Usual care (mean SD) *n* = 67
Baseline	7.6 (1.7, 196)	6.9 (1.73)	6.5 (2.13)
EOT^1^	5.4 (2.6, 196)	4.1 (2.01)	5.5 (2.61)
Post-tx 4	4.9 0 (2.5, 154)	n/a	n/a
Final follow-up^2^	5.2 (2.56, 135)	3.7 (2.16)	5.5 (2.45)
Change: final follow-up minus baseline	− 2.4	− 3.2	− 1

^1^EOT is after the 8th treatment for NADA and 9 weeks after randomisation for CBT study

^2^Final follow-up is 18 weeks post EOT for NADA and 26 weeks post randomisation for CBT study

We also compared our HFNS frequency with the CBT study, which showed the NADA service users had a greater decrease in frequency (− 23 HFNS/week) than those practising CBT (− 16/week) and usual care (− 7/week) at the final follow-up points.

### Associated menopausal symptoms

Our data show improvements in a range of symptoms associated with the menopause at EOT, including depressed mood, somatic symptoms, memory/concentration, vasomotor symptoms, anxiety/fears, and sleep problems. Improvements were maintained at the 4-week follow-up for all but depressed mood and memory/concentration. However, benefit at 18 weeks post-treatment was sustained for anxiety/fears and vasomotor symptoms only. The benefit at EOT accords with results of a systematic review of six RCTs (207 participants) that reported on validated menopausal scales [20], but falls short of the 3 months reported in another systematic review of the maintenance effect of acupuncture on menopause symptoms (five studies analysed) [17].

### Comparison with our previous NADA research data

Finally, this service was set up on the model of our previous research. In comparing the outcomes of the service and research, we found that overall responses observed in the service users compared favourably with the results collected more rigorously from the research.

### Recommendations

This evaluation comes at a time when the service is planning to update its scope and aims. In shaping a new service, we have discussed whether to dispense with the hot flush diaries and rely on the HFRS, which would be easier for service users to complete. However, clinically we have found that keeping hot flush diaries often helps women to fully realise the extent of their HFNS, as well as helping them to identify patterns, which may provide insight and a measure of control. We recommend completing diaries for a week rather than a fortnight.

We also recommend replacing the WHQ with the hot flash related daily interference scale (HFRDIS). This 10-item questionnaire measures impact of HFNS on daily activities and quality of life in the past week [32]. This much shorter questionnaire may simplify data completion for women, some of whom struggled with the paperwork in the service and would also simplify analysis.

## Conclusion

This first analysis of a long-term acupuncture service for HFNS and first long-term report of outcomes for NADA compares favourably with outcomes for other studies for improving consequences of adjuvant hormonal treatments for breast cancer, including frequency of HFNS and HFNS as a problem, as well as menopausal health–related quality of life. Furthermore, this proved to be a sustainable intervention over the long term for all parties, including managers, funders, therapists, and breast cancer survivors. In day-to-day clinical practice, NADA appears to be a safe, effective intervention for women who do not wish to use pharmacological means to control their HFNS.
